# Supplementary material for: Moderately Inducing Autophagy Reduces Tertiary Brain Injury after Perinatal Hypoxia-Ischemia
Source: Cells. 2021 Apr 14;10(4):898. doi: 10.3390/cells10040898 (PMC8070811; doi:10.3390/cells10040898)
Supplement: Supplementary file 1 [file cells-10-00898-s001.zip › cells-1053876-supplementary-new.docx]

**
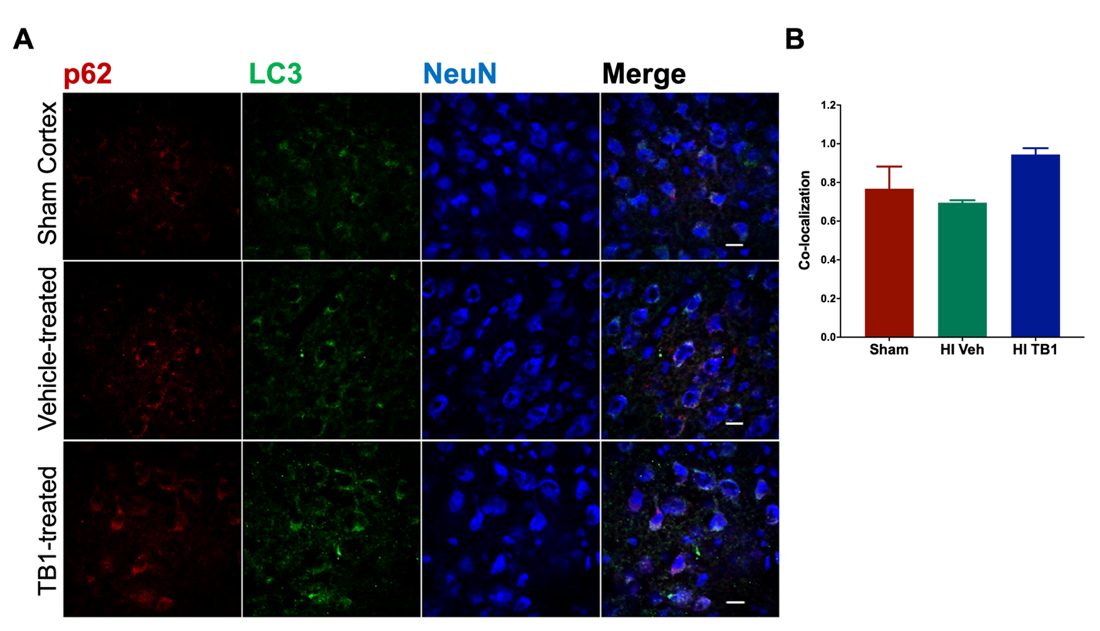
**
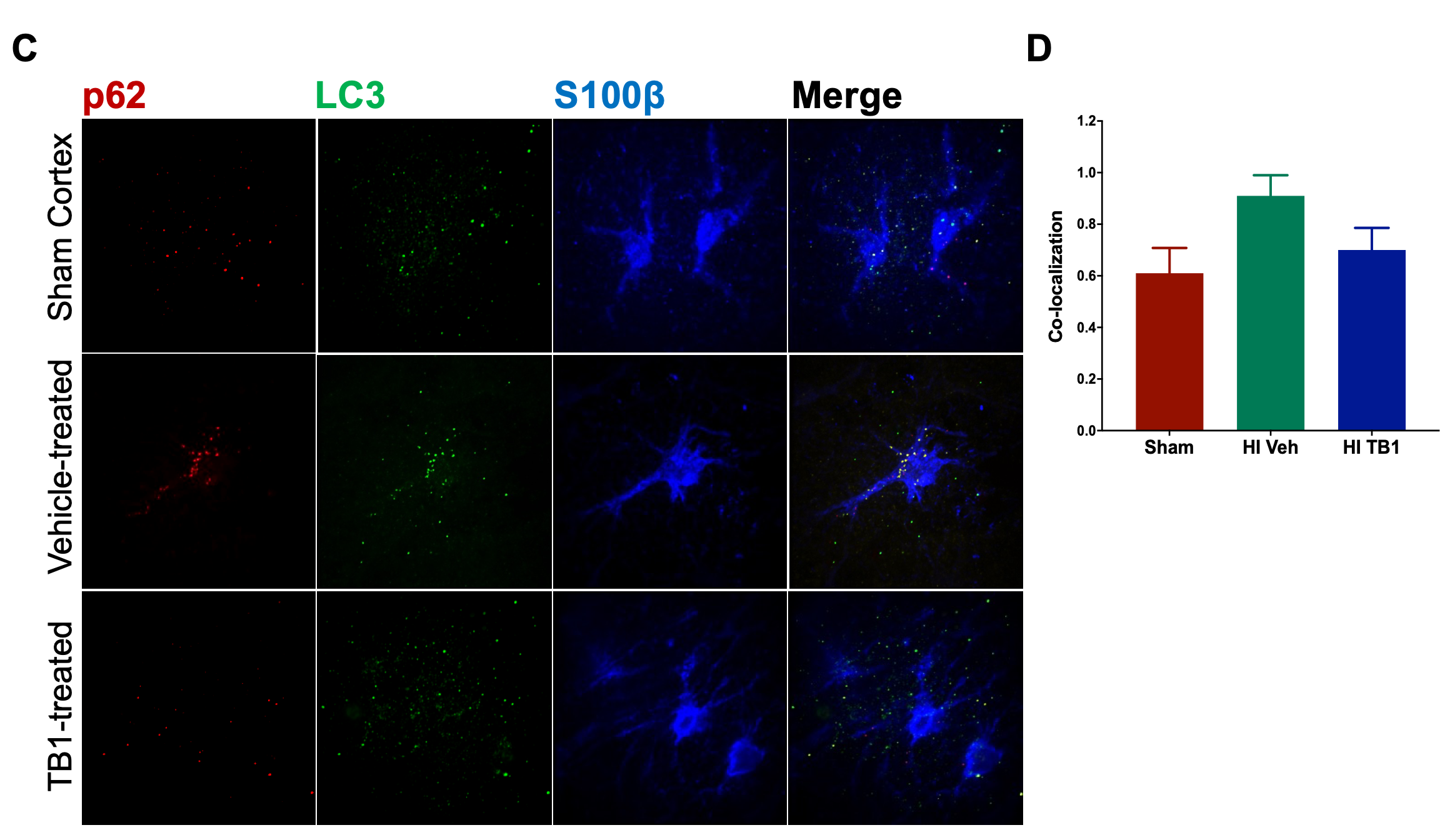


**Figure S1.** Systemic Tat-Beclin1 administration increases P62 and LC3 colocalization in neurons but not in astrocytes up to 48 h after injection. Three days after HI, rat pups were injected i.p. with Tat-Beclin1 (15mg/kg) or vehicle (PBS). 48 h after injection, samples of the injured forebrain were prepared for immunofluorescence analysis. (**A**) Representative cortical neurons in the ischemic penumbra of injury with Sham neocortex as control stained with anti-p62 (red), anti-LC3 (green), and anti-NeuN (blue) markers. (**B**) Manders’ colocalization coefficient (M1) for the fractional overlap of p62 signal in compartments containing LC3 signal for the neocortex. (**C**) Representative astrocytes in the ischemic penumbra with Sham neocortex as control stained with anti-p62 (red), anti-LC3 (green), and anti-S100β (blue) markers. (**D**) M1 colocalization coefficient for the fractional overlap of p62 signal in compartments containing LC3 signal for the neocortex. Values represent averages ± SEM. n = 3/group.
